# Comparative Predictive Accuracy of ASA, POSSUM, and NSQIP Risk Scoring Systems in Major Abdominal Surgeries: A Systematic Review

**DOI:** 10.7759/cureus.85572

**Published:** 2025-06-08

**Authors:** Vasudeva Lam, Muhammad Muaz Loon, Minaa Alrawe, Ismail S Abougendy, Muhammad Ali

**Affiliations:** 1 General Surgery, Queens Hospital, Romford, GBR; 2 General Surgery, Lady Hardinge Medical College, Delhi, IND; 3 General Surgery, Barking, Havering and Redbridge University Hospitals NHS Trust, London, GBR; 4 Emergency Department, British Hospital, Cairo, EGY; 5 General Surgery, Misr University for Science and Technology, Cairo, EGY; 6 General Surgery, Nishtar Medical University, Multan, PAK

**Keywords:** asa score, frailty index, major abdominal surgery, mortality prediction, nsqip, possum, postoperative outcomes, preoperative assessment, risk stratification, surgical risk prediction

## Abstract

This systematic review evaluates and compares the predictive accuracy of three commonly used surgical risk scoring systems in the context of major abdominal surgeries: American Society of Anesthesiologists (ASA) score, Physiological and Operative Severity Score for the enUmeration of Mortality and Morbidity (POSSUM), including its variants, and American College of Surgeons National Surgical Quality Improvement Program (NSQIP) calculator. A comprehensive literature search was conducted across multiple databases, identifying 10 eligible studies that assessed these models in various surgical settings, including elective colorectal resections, emergency laparotomies, and IBD-related procedures. The findings revealed that NSQIP-based models, especially those incorporating frailty indices, demonstrated better calibration and discrimination in elective surgeries. POSSUM and P-POSSUM provided moderate to high predictive accuracy in emergency settings, though they frequently overestimated mortality, particularly in laparoscopic cases. The ASA score, while simple and widely used, showed moderate predictive value but limited precision in complex or high-risk scenarios. Significant heterogeneity in study design, population characteristics, and outcome reporting precluded meta-analysis. However, narrative synthesis confirmed that the performance of each scoring system is context-dependent and that no single model demonstrated consistent superiority across all surgical types. These results underscore the importance of tailored risk assessment strategies and highlight the need for recalibration, external validation, and integration of newer predictive variables in surgical risk modeling.

## Introduction and background

Accurately predicting surgical risk is a cornerstone of modern perioperative care, particularly in major abdominal surgeries, which are often associated with considerable morbidity and mortality [1]. Preoperative risk stratification tools aid in clinical decision-making, inform patient consent, guide postoperative resource allocation, and support comparative performance benchmarking across institutions. Among the numerous scoring systems developed to assess surgical risk, the American Society of Anesthesiologists (ASA) [2] Physical Status Classification, the Physiological and Operative Severity Score for the enUmeration of Mortality and Morbidity (POSSUM) [3] along with its variants (such as P-POSSUM and CR-POSSUM), and the American College of Surgeons National Surgical Quality Improvement Program (NSQIP) [4] calculator have been widely adopted in surgical practice.

Each of these scoring systems was developed with distinct methodologies and has varying scopes and applications. The ASA score, one of the oldest and simplest tools, is subjective and relies on an anesthesiologist’s clinical judgment regarding the patient’s physical status. While convenient, it lacks the granularity to account for surgical complexity and specific comorbidities [5]. In contrast, the POSSUM and its variants are more comprehensive, incorporating both physiological and operative parameters to generate mortality and morbidity estimates, yet their accuracy has been debated, especially across different surgical populations. NSQIP, on the other hand, is a data-driven model derived from a large national database and integrates numerous preoperative factors to predict individualized risk estimates [6]. Its utility has been demonstrated in various surgical domains, but its accessibility and dependence on specific datasets limit its universality.

Despite their widespread use, there remains considerable variability in the reported predictive performance of ASA, POSSUM, and NSQIP scoring systems, particularly across diverse populations undergoing major abdominal surgery, such as colorectal resections, emergency laparotomies, and inflammatory bowel disease-related operations. While several individual studies have attempted to validate and compare these models, inconsistencies in patient selection, surgical urgency, outcome definitions, and methodological rigor have led to heterogeneous findings. Notably, despite the clinical relevance of these tools, no comprehensive systematic review or meta-analysis to date has compared their predictive accuracy across both elective and emergency abdominal surgeries. To address this gap, the objective of this systematic review was to evaluate and compare the predictive accuracy of ASA, POSSUM (including its variants), and NSQIP scoring systems in major abdominal surgeries, with the goal of informing clinical decision-making and enhancing risk-stratification practices.

## Review

Materials and methods

Search Strategy

This systematic review followed the Preferred Reporting Items for Systematic Reviews and Meta-Analyses (PRISMA) guidelines [7]. Although the review was not registered with PROSPERO, it was conducted in strict adherence to established methodological standards to ensure transparency, reproducibility, and rigor. A systematic and comprehensive search of the literature was conducted across major databases, including PubMed, Scopus, and Web of Science to identify studies assessing the predictive performance of the ASA, POSSUM, and NSQIP scoring systems in the context of major abdominal surgeries. The search included combinations of relevant terms such as “ASA score,” “American Society of Anesthesiologists,” “POSSUM,” “P-POSSUM,” “NSQIP,” “surgical risk,” “abdominal surgery,” “colorectal surgery,” “emergency laparotomy,” “mortality,” and “postoperative complications.” Boolean operators were used to combine terms appropriately. The search was restricted to English-language articles involving human subjects, and reference lists of included articles were manually reviewed to identify any additional eligible studies.

Eligibility Criteria and PICO Framework

Studies were selected based on predefined eligibility criteria aligned with the objectives of this systematic review. We included original, peer-reviewed articles that evaluated the predictive performance of the ASA score, POSSUM or its variants (such as P-POSSUM or CR-POSSUM), and NSQIP-based scoring systems in adult patients undergoing major abdominal surgery, including but not limited to colorectal resections, emergency laparotomies, and gastrointestinal procedures. Eligible study designs included retrospective and prospective cohort studies, comparative analyses, and post hoc evaluations of clinical trials that reported relevant outcomes such as postoperative mortality, morbidity, or major complications. Studies were excluded if they lacked outcome data pertinent to predictive performance (e.g., receiver operating characteristic (ROC) curves, odds ratios (ORs), sensitivity/specificity), did not evaluate at least one of the specified scoring systems, involved pediatric populations, or were non-English language publications, reviews, editorials, or conference abstracts.

Although this review evaluates prognostic tools rather than therapeutic interventions, we utilized a modified PICO framework [8] to maintain clarity and consistency in study selection. In this context, the Population (P) comprised adult patients undergoing major abdominal surgeries. The "Intervention" (I) was operationalized as the application of one of the three predictive models--ASA, POSSUM, or NSQIP. The Comparison (C) involved either direct comparison between these tools or their alignment with observed clinical outcomes. The Outcome (O) centered on the prediction of 30-day postoperative mortality and major complications, including infection, reoperation, or ICU admission. While a prognostic-specific framework such as PICOTS (Population, Index test, Comparator, Outcome, Timing, Setting) is more comprehensive, the simplified PICO format allowed structured and practical categorization of study elements while implicitly considering timing and setting through data extraction. This approach facilitated the systematic identification and synthesis of studies assessing the comparative predictive accuracy of widely used surgical risk assessment tools.

Data Extraction

Data from each included study were systematically extracted using a standardized template developed a priori to ensure consistency and reduce bias. Two independent reviewers screened titles and abstracts for relevance, followed by full-text review to assess eligibility based on predefined inclusion criteria. Disagreements at either stage were resolved through discussion or by involving a third reviewer. For each study, we extracted relevant information including study design, sample size, surgical context, scoring system(s) evaluated (ASA, POSSUM, NSQIP), outcome measures (e.g., 30-day mortality, postoperative complications), and predictive performance metrics such as AUC, odds ratios (ORs), sensitivity, and specificity. When numeric data were not directly reported, values were extracted from graphs using WebPlotDigitizer (version 4.5, http://arohatgi.info/WebPlotDigitizer). Risk of bias and methodological quality were independently assessed using validated tools appropriate for each study design. Extracted data were compiled into a summary table to facilitate structured comparison across studies.

Quality Assessment

To evaluate the methodological quality and risk of bias of the included studies, we used validated assessment tools appropriate to each study design. The NIH Quality Assessment Tool for Observational Cohort and Cross-Sectional Studies was applied to retrospective and prospective observational studies [9]. For the prospective database validation study, we used the Prediction model Risk Of Bias assessment Tool (PROBAST) [10], and for the post hoc analysis of a randomized controlled trial, the Risk Of Bias In Non-randomized Studies - of Interventions (ROBINS-I) tool was utilized [11]. Two reviewers independently conducted the quality assessment, with disagreements resolved through discussion or consultation with a third reviewer. The criteria evaluated included study design, sample selection, outcome measurement, statistical analysis, and control of confounding.

Data Analysis and Synthesis

Given the heterogeneity in study designs, patient populations, scoring systems, and reported outcomes, a narrative synthesis approach was employed rather than a formal meta-analysis. The predictive accuracy of each scoring system--ASA, POSSUM (including its variants), and NSQIP--was evaluated across studies based on reported AUC values, regression estimates, and calibration data. Studies were grouped and analyzed according to the risk model used and the surgical context (e.g., elective colorectal surgery vs emergency laparotomy). Comparative findings between models, where available, were highlighted to assess relative performance. Emphasis was placed on identifying trends, consistencies, and methodological limitations across studies, as well as exploring potential modifiers of predictive performance such as surgical urgency, patient age, and comorbidity burden.

Results

Study Selection Process

**Figure 1:**
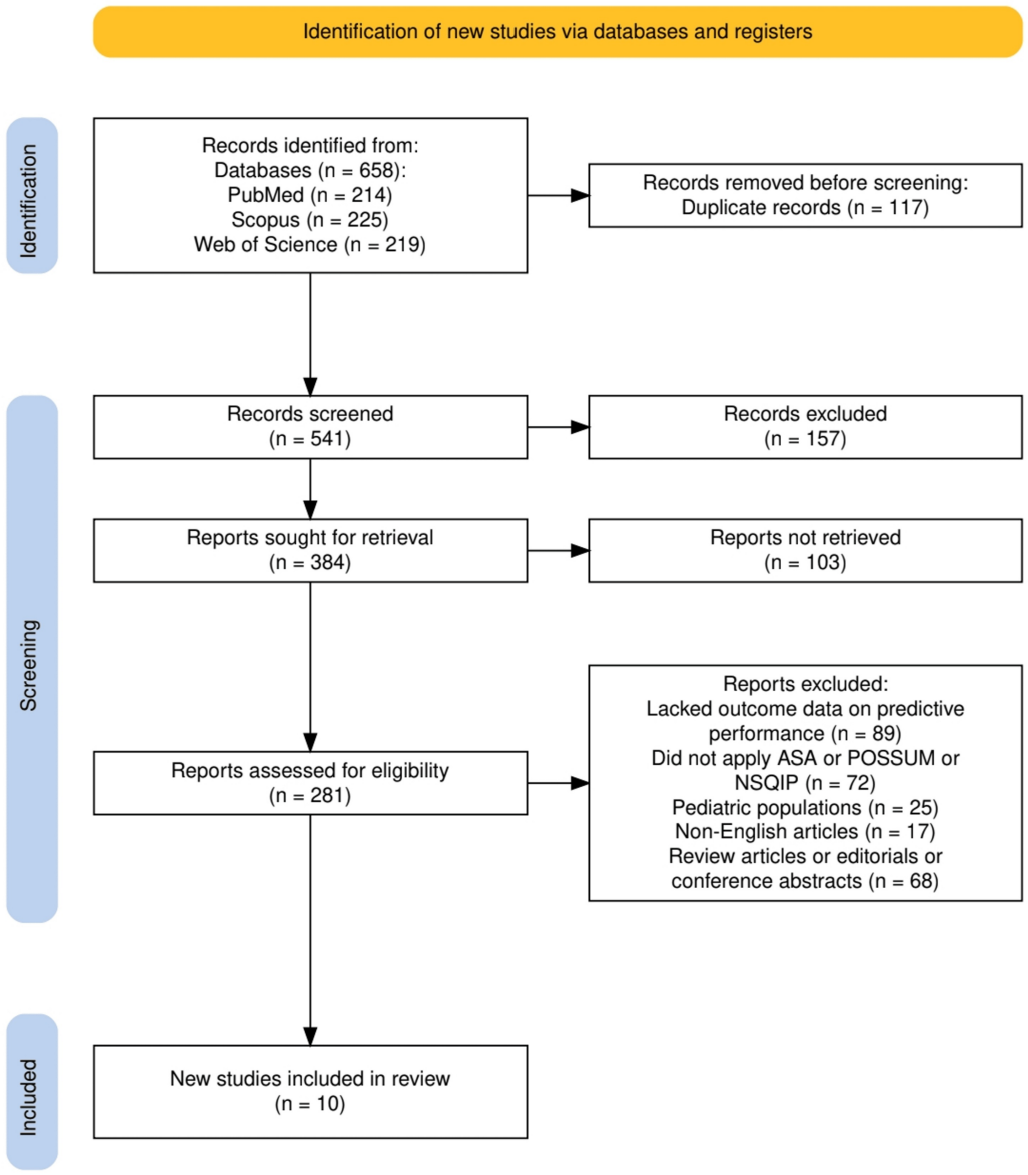
The PRISMA flowchart represents the study selection process. PRISMA: Preferred Reporting Items for Systematic reviews and Meta-Analyses

The study selection process is illustrated in Figure 1. A total of 658 records were identified through electronic database searches, including 214 from PubMed, 225 from Scopus, and 219 from Web of Science. After the removal of 117 duplicate records, 541 records were screened based on titles and abstracts. Of these, 157 were excluded as irrelevant to the research question. Full texts of 384 articles were sought for retrieval; however, 103 articles could not be accessed due to paywall restrictions, broken institutional links, or missing archival records. We attempted to obtain these through interlibrary loan services and contacted corresponding authors for 48 potentially relevant articles, but most remained inaccessible due to lack of response or repository limitations. After excluding these, 281 full-text articles were assessed for eligibility. Following detailed evaluation, 271 articles were excluded based on predefined criteria: 89 lacked relevant outcome data, 72 did not evaluate ASA, POSSUM, or NSQIP, 25 involved pediatric populations, 17 were non-English, and 68 were reviews, editorials, or conference abstracts. Ultimately, 10 studies met all inclusion criteria and were incorporated into the final evidence synthesis.

Characteristics of the Selected Studies

The key characteristics of the ten studies included in this systematic review are summarized in Table 1. The selected studies encompassed a range of retrospective, prospective, and observational cohort designs, with sample sizes varying from 193 to over 116,000 patients. Surgical procedures primarily involved major abdominal operations, including elective colorectal resections, emergency laparotomies, and inflammatory bowel disease-related surgeries. The scoring systems evaluated across these studies included ASA, various forms of POSSUM (P-POSSUM, CR-POSSUM), and NSQIP-based models, including the modified frailty index (mFI-5). Comparators varied depending on study objectives and included albumin levels, operative approach, comorbidity indices, and direct comparisons of predicted versus observed mortality. The primary outcomes assessed were 30-day mortality, major postoperative complications, and, in some cases, intensive care unit admission or length of stay. Predictive performance was reported using metrics such as the area under the ROC curve (AUC) and odds ratios, with results demonstrating considerable variability. While some studies reported strong discrimination by certain models, others highlighted limitations in calibration or performance in specific subgroups, underscoring the need for context-specific application of these scoring tools.

**Table 1:** Summary of included studies evaluating the predictive performance of ASA, POSSUM (and its variants), and NSQIP scoring systems in major abdominal surgery. Comparator variables that are not formal scoring systems (e.g., albumin levels, operative approach) were included when used to adjust or contextualize risk model performance. ACS, American College of Surgeons; APACHE II, Acute Physiology and Chronic Health Evaluation II; ASA, American Society of Anesthesiologists; AUC, area under the ROC curve; ROC, receiver operating characteristic; CCI, Charlson Comorbidity Index; IBD, inflammatory bowel disease; IRR, incidence rate ratio; LOS, length of stay; mFI-5, Modified 5-item Frailty Index; NELA, National Emergency Laparotomy Audit; NSQIP, National Surgical Quality Improvement Program; P-POSSUM, Portsmouth Physiological and Operative Severity Score for the enUmeration of Mortality and Morbidity; RCT, randomized controlled trial; SDOS, Surgical Death Observation Score

Study (Author, Year)	Study Design	Sample Size	Type of Surgery	Scoring System(s) Evaluated	Comparator(s)	Primary Outcome(s)	Predictive Performance	Key Findings
Nguyen et al., 2019 [12]	Retrospective cohort	10,913	IBD-related major abdominal surgery	NSQIP	Albumin levels (used for stratification, not model comparison)	30-day mortality, infectious and non-infectious complications	OR not reported; mortality rates stratified by albumin levels	Severe hypoalbuminemia linked to increased mortality and complications
Keller et al., 2018 [13]	Retrospective observational	355	Elective colorectal surgery	NSQIP, ACS Risk Calculator	Observed outcomes	Any complication, readmission, mortality	AUC < 0.60 for all except NSQIP mortality	NSQIP and ACS tools correlated but poorly predicted complications; NSQIP mortality accurate
Keller et al., 2020 [14]	Retrospective cohort	412	Elective colorectal surgery	mFI-5 (NSQIP-derived)	Frailty score groups (0 vs 1 vs 2+)	Major morbidity/mortality, minor complications	OR = 4.62 for mFI-5 ≥2; ROC poor unless adjusted	mFI-5 associated with complications but limited standalone predictive value
Ah et al., 2019 [15]	Retrospective cohort	209	Emergency laparotomy (geriatric)	P-POSSUM	Adjusted using osteopenia, ASA, age, malignancy	30-day mortality	AUC: 0.59 (raw), 0.83 (adjusted); IRR = 1.58	P-POSSUM alone poorly predictive; model improved with adjustment
Sharrock et al., 2017 [16]	Observational cohort	193	Emergency laparotomy (≥70 yrs)	P-POSSUM, ASA	Included lactate and ASA in model	30-day mortality	AUC: P-POSSUM = 0.784, ASA = 0.771, Lactate = 0.705	Moderate predictive value; frailty may improve risk discrimination
Darbyshire et al., 2022 [17]	Prospective database analysis (NELA)	116,396 (P-POSSUM), 46,935 (NELA)	Emergency bowel surgery (laparoscopic vs open)	P-POSSUM, NELA	Operative approach (subgroup analysis)	30-day mortality	P-POSSUM: 0.801–0.836; NELA: 0.811–0.862	Both overpredicted mortality in laparoscopic surgery despite good discrimination
Lodha et al., 2025 [18]	Prospective observational	238	Emergency laparotomy	P-POSSUM, NELA	Predicted vs observed mortality	30-day mortality	AUC: NELA = 0.699, P-POSSUM = 0.687; NELA Sensitivity = 73.9%, Specificity = 45.6%	NELA more accurate than P-POSSUM for mortality prediction
Ragg et al., 2009 [19]	Prospective observational	887	Major colorectal surgery	ASA	Evaluated comorbidity, urgency, and rectal surgery	Mortality, major morbidity	AUC (mortality): 0.87; morbidity: 0.69	ASA Grade III–V and urgency predicted worse outcomes
Hansted et al., 2020 [20]	Post hoc analysis of RCT	885	Emergency abdominal surgery	ASA, APACHE II, CCI	Observed outcomes	30-/90-day mortality, ICU admission	AUC: APACHE II = 0.72 (30-day); ASA similar; ICU prediction poor	ASA comparable to APACHE II for mortality; poor ICU prediction
Dekker et al., 2012 [21]	Multivariate cohort analysis	2,204	Colorectal cancer surgery (Stage I–III)	ASA, CCI, SDOS	Comorbidity-adjusted vs non-adjusted models	30-day mortality, complications, LOS	ROC marginally improved by adding comorbidities	ASA and comorbidity scores were independent risk factors; added limited predictive gain

Quality Assessment

The methodological quality of the included studies was evaluated using appropriate appraisal tools based on study design, as summarized in Table 2. The NIH Quality Assessment Tool [9] for Observational Cohort Studies was applied to the majority of the studies, while the PROBAST tool [10] was used for the prospective database validation study, and the ROBINS-I tool [11] was applied to the post hoc analysis of a randomized trial. Of the ten studies assessed, four were judged to have a low risk of bias, demonstrating sound methodology, transparent reporting, and appropriate statistical analysis. These studies generally involved prospective designs, robust databases, or adjusted analyses that enhanced internal validity. The remaining six studies were rated as having some concerns, primarily due to retrospective designs, limited control for confounding, potential selection bias, or lack of external validation. Nonetheless, all studies were deemed to provide relevant and usable data for synthesis, contributing meaningful insights into the predictive value of ASA, POSSUM, and NSQIP-based scoring systems in major abdominal surgery.

**Table 2:** Overview of study design, applied quality assessment tools, and risk of bias judgments for the studies included in this systematic review. AUC, area under the receiver operating characteristic curve; NIH, National Institutes of Health; NSQIP, National Surgical Quality Improvement Program; P-POSSUM, Portsmouth Physiological and Operative Severity Score for the enUmeration of Mortality and Morbidity; NELA, National Emergency Laparotomy Audit; PROBAST, Prediction model Risk Of Bias ASsessment Tool; RCT, randomized controlled trial; ROBINS-I, Risk Of Bias In Non-randomized Studies of Interventions; ROC, receiver operating characteristic; Sens, sensitivity; Spec, specificity.

Study (Author, Year)	Study Design	Tool Used	Risk Judgment	Score or Domain Summary	Justification
Nguyen et al., 2019 [12]	Retrospective cohort	NIH	Some concerns	9/14 “Yes” items; unclear confounding & blinding	Strong NSQIP data, but retrospective design limits control of confounders and lacks prospective exposure assessment.
Keller et al., 2018 [13]	Retrospective observational	NIH	Some concerns	8/14 “Yes”; no adjustment for confounding	Single-institution data, appropriate AUC usage, but no multivariable adjustment.
Keller et al., 2020 [14]	Retrospective cohort	NIH	Low risk	12/14 “Yes”; adjusted analyses present	Clear outcome/exposure assessment, good internal validity despite modest sample size.
Ah et al., 2019 [15]	Retrospective cohort	NIH	Some concerns	9/14 “Yes”; selection bias possible	Used regression appropriately but had limited confounding control and potential selection bias.
Sharrock et al., 2017 [16]	Observational cohort	NIH	Low risk	13/14 “Yes”; strong variable control	Transparent reporting, robust use of ASA and P-POSSUM; well-conducted.
Darbyshire et al., 2022 [17]	Prospective audit database	PROBAST	Low risk	Low risk across all 4 domains	Based on high-quality NELA database, with rigorous external validation and calibration.
Lodha et al., 2025 [18]	Prospective observational	NIH	Low risk	11/14 “Yes”; minor missing data issues	Prospective design, complete reporting of ROC/Sens/Spec, minimal concerns.
Ragg et al., 2009 [19]	Prospective observational	NIH	Some concerns	8/14 “Yes”; lacks external validation	Model overfitting risk and no blinding; otherwise adequate reporting.
Hansted et al., 2020 [20]	Post hoc analysis of RCT	ROBINS-I	Low risk	Low risk in 6/7 domains; moderate risk in “Selection of reported results”	Good comparability with other tools; outcome measurement solid; minor reporting limitation.
Dekker et al., 2012 [21]	Multivariate cohort analysis	NIH	Some concerns	9/14 “Yes”; outcome assessor blinding unclear	Strong statistical modeling, but observational design limits causal inference; some reporting gaps.

Discussion

This systematic review evaluated the comparative predictive performance of three widely used preoperative risk assessment tools in major abdominal surgeries: ASA, POSSUM (including P-POSSUM and its variants), and NSQIP-based models. NSQIP-based models consistently demonstrated favorable outcome calibration, particularly in elective settings, due to their incorporation of granular perioperative variables such as frailty and hypoalbuminemia [12,13]. For instance, severe hypoalbuminemia was associated with significantly increased 30-day mortality in both Crohn’s disease (0.7% vs. 0.2%, p < 0.05) and ulcerative colitis (5.6% vs. 0.1%, p < 0.01), highlighting the prognostic value of nutritional status [12]. Similarly, the mFI-5 frailty score-derived from NSQIP-was a significant predictor of postoperative complications: patients with a score ≥2 had over four times the odds of major complications compared to lower-risk groups (OR 4.62; 95% CI: 1.44-14.78; p = 0.010), underscoring the importance of frailty screening in elderly or comorbid patients [14].

POSSUM and P-POSSUM models demonstrated high discriminatory ability in emergency surgery cohorts. For example, the National Emergency Laparotomy Audit (NELA) study reported excellent predictive performance with c-statistics ranging from 0.801-0.836 for P-POSSUM and 0.811-0.862 for NELA, although both models consistently overestimated mortality in laparoscopic cases [17]. This indicates that while these scores remain robust, recalibration is warranted for use in minimally invasive settings. Another study reported that P-POSSUM's AUC improved markedly from 0.59 (unadjusted) to 0.83 (adjusted) when incorporating covariates such as age and comorbidities (Incidence Rate Ratio = 1.58; p = 0.004), illustrating that its accuracy improves when individualized risk factors are considered [15]. Clinically, this supports the adaptable use of POSSUM-based tools in diverse operative scenarios.

The ASA score, though widely adopted for its simplicity, exhibited moderate predictive utility. In a cohort of 887 patients, ASA Grades III-V were associated with increased postoperative mortality (AUC = 0.87) and major morbidity (AUC = 0.69), validating its value in preoperative risk stratification [19]. However, in a separate study involving 885 patients undergoing emergency abdominal surgery, ASA and APACHE II had comparable predictive accuracy for 30-day mortality (AUC = 0.72), but both showed poor discrimination for ICU admission (AUC = 0.65), highlighting their limitations in forecasting more complex postoperative needs [20].

Taken together, these findings suggest that NSQIP models are best suited for elective surgery planning, offering good calibration in real-world data; P-POSSUM is valuable for risk estimation in emergency or high-risk patients when combined with patient-specific adjustments; and ASA remains a convenient but limited screening tool. The selection of a predictive model should consider the clinical context, available data, and need for precision versus practicality.

The findings of this review are largely consistent with previous literature comparing the performance of surgical risk stratification tools across different operative contexts [22]. Several earlier reviews have affirmed that NSQIP-based models, particularly those incorporating frailty indices, outperform traditional scoring systems in elective surgeries due to their individualized, data-rich framework. Our findings reinforce this trend, with NSQIP showing more accurate mortality prediction in elective colorectal procedures, as evidenced by Keller et al. [14], where NSQIP outperformed the ACS calculator in mortality prediction despite poor performance in identifying complications (AUC < 0.60 for most outcomes). Conversely, P-POSSUM and NELA scores were more frequently evaluated in emergency settings, where they demonstrated strong discrimination (c-statistics > 0.80), but often lacked calibration in laparoscopic surgeries, a limitation also noted in large-scale audit reports such as the UK’s NELA [23]. Studies have previously highlighted that POSSUM tends to overestimate mortality, especially in lower-risk cases and minimally invasive procedures, which was also observed in our synthesis. The ASA score, while convenient and widely adopted, has repeatedly been criticized in the literature for its subjectivity and inter-observer variability [24]. Despite this, some studies report comparable discrimination to more complex tools in high-risk cohorts, echoing our finding that ASA achieved an AUC of 0.87 in mortality prediction among colorectal cancer patients. These comparisons suggest that while all three tools have utility, their performance is highly dependent on clinical context, surgical urgency, and patient characteristics--underscoring the importance of tailored model selection in perioperative care.

The findings of this review carry important clinical implications for surgical risk stratification and preoperative decision-making in major abdominal surgeries. Accurate risk prediction is essential for guiding operative planning, informing patient consent, and optimizing resource allocation, particularly in high-risk populations. The evidence suggests that NSQIP-based models, with their inclusion of granular, patient-specific data and optional frailty indices, are best suited for elective surgical settings, especially in colorectal and IBD-related procedures [25], where they consistently demonstrate reliable performance in predicting mortality and complications. In contrast, POSSUM and P-POSSUM models appear to offer more value in emergency surgeries [26], although their tendency to overestimate risk--particularly in laparoscopic procedures--necessitates cautious interpretation. The ASA score, while widely used due to its simplicity and accessibility, should not be used in isolation for complex surgical cases, as its subjectivity limits its precision [27]. Integrating more nuanced tools like NSQIP or supplementing ASA with objective parameters may provide a more balanced risk profile. Ultimately, utilizing the most appropriate model for the clinical context can enhance patient counseling, guide surgical triage, and support the development of evidence-based, individualized perioperative care pathways that improve both short- and long-term outcomes.

This systematic review possesses several strengths that enhance its credibility and relevance. The study adhered rigorously to the PRISMA guidelines, ensuring a transparent and methodologically sound approach to literature selection, data extraction, and synthesis. A comprehensive search strategy was employed across multiple major databases--PubMed, Scopus, and Web of Science--using well-defined search terms and Boolean operators, which maximized sensitivity and minimized omission of relevant studies. Additionally, the use of clear eligibility criteria and structured application of appropriate quality assessment tools tailored to each study design allowed for critical appraisal of the included evidence. However, certain limitations must be acknowledged. The included studies demonstrated significant heterogeneity in terms of design, patient populations, surgical procedures, and outcome measures, precluding the possibility of conducting a formal meta-analysis. Differences in how predictive performance was reported (e.g., AUC vs OR) further complicated direct quantitative comparisons. Moreover, the review was limited to English-language publications, introducing the possibility of language bias, and the reliance on published literature may have led to publication bias, favoring studies with positive or statistically significant findings. Despite these constraints, the review provides valuable insight into the comparative utility of widely used surgical risk assessment tools.

Future research should focus on the recalibration and refinement of existing surgical risk scoring systems to align with evolving clinical practices, particularly in the context of minimally invasive techniques [22] and enhanced recovery protocols. While tools like NSQIP and POSSUM have shown utility, their predictive accuracy may diminish when applied to modern surgical populations, necessitating periodic updates and context-specific adjustments. Additionally, there is a pressing need for external validation of these models in underrepresented settings, including low- and middle-income countries (LMICs), where healthcare infrastructure, perioperative risk profiles, and case-mix differ significantly from those in high-income settings. Similarly, elderly patients and those undergoing laparoscopic procedures remain inadequately represented in current validation cohorts, limiting the generalizability of these tools. The integration of frailty indices, as exemplified by the mFI-5, offers a promising step toward individualized risk prediction. Looking ahead, the incorporation of machine learning algorithms and real-time electronic health record data may enable the development of dynamic, AI-driven models that surpass static scoring systems in accuracy and adaptability, ultimately transforming perioperative risk assessment into a more personalized and responsive process.

## Conclusions

This systematic review provides a comprehensive comparative evaluation of three widely used surgical risk stratification tools--ASA, POSSUM, and NSQIP--in the context of major abdominal surgeries. The findings highlight significant variability in the predictive accuracy of these models, underscoring the importance of selecting scoring systems based on clinical context, surgical urgency, and patient-specific factors. While NSQIP-based models, particularly those incorporating frailty indices, demonstrated enhanced predictive utility in elective surgeries, POSSUM showed moderate-to-good discrimination in emergency settings when adjusted for relevant covariates. The ASA score, although widely adopted, exhibited limited precision when used in isolation. By synthesizing current evidence across a diverse range of study designs and surgical populations, this review not only informs clinical practice but also identifies critical gaps in risk prediction that warrant further investigation. Importantly, this study emphasizes the need for dynamic, context-sensitive, and data-integrated approaches to preoperative risk assessment, which are essential for improving surgical outcomes, optimizing resource allocation, and guiding informed patient care in both high- and low-resource settings.
